# Resilience, emotion regulation, and interpreting learning engagement: cognitive reappraisal as a mediator rather than expressive suppression

**DOI:** 10.3389/fpsyg.2026.1916373

**Published:** 2026-09-03

**Authors:** Wenting Yu, Chenggang Wu

**Affiliations:** 1 School of English Studies, Shanghai International Studies University, Shanghai, China; 2 Key Laboratory of Multilingual Education with AI, School of Education, Shanghai International Studies University, Shanghai, China; 3 Institute of Linguistics, Shanghai International Studies University, Shanghai, China

**Keywords:** resilience, emotion regulation, cognitive reappraisal, expressive suppression, interpreting learning engagement

## Abstract

Drawing on positive psychology concepts, this cross-sectional study investigates associations among resilience, emotion regulation (specifically cognitive reappraisal [CR] and expressive suppression [ES]), and interpreting learning engagement. A total of 306 interpreting learners from five universities in China completed validated questionnaires. A theoretically specified associative mediation model treated resilience as a relatively distal adaptive resource, CR and ES as more proximal emotion regulation processes, and engagement as the learning correlate. The structural model was estimated with the DWLS estimator with Bollen-Stine bootstrapping (1,000 resamples) to examine indirect effects and confidence intervals. The findings indicated that resilience and CR were positively associated with and could predict interpreting learning engagement across all four dimensions (adjusted *R*^2^ ranged from 10.7 to 16.8%). In the specified model, CR partially mediated the link between resilience and interpreting learning engagement (proportion mediated ranged from 25.6 to 50%), whereas ES exhibited neither a significant correlation with resilience and engagement nor a mediating role in this relationship. These results highlight CR as an adaptive emotion regulation strategy relevant to engagement in the cognitively and emotionally demanding context of interpreter training. However, because all variables were measured concurrently, the predictive and mediation results should be interpreted statistically rather than causally. Longitudinal and experimental studies are needed to establish temporal direction and evaluate CR-focused pedagogical practices.

## 1 Introduction

Interpreting is a cognitively demanding skill that requires concurrent listening, comprehension, reformulation, and production across languages under severe time pressure, placing considerable demands on learners’ attention, working memory, executive control, and language competence (Gile, 1995; Moser-Mercer, 2008; Seeber, 2011). Accordingly, interpreter education and research have traditionally centered on cognitive and linguistic competencies such as memory, language proficiency, and executive control (Dong, 2018; Johnson, 2001; Kurz, 2003; Liu, 2008). However, psychological factors that may be associated with learners’ development and sustained involvement throughout interpreting training have received comparatively limited attention (Cai et al., 2023). This is particularly important because learning to interpret is frequently accompanied by frustration and anxiety, which may coincide with difficulties in learning progress. As Kurz (2003) noted, interpreting learners often experience high physiological stress in classroom settings. Such psychological challenges may also be associated with lower sustained interest and active participation, which are key aspects of learning engagement.

Learning engagement is defined as students’ goal-directed involvement in learning tasks (Hiver et al., 2021) and is a key predictor of educational attainment and positive learning outcomes (Kahu and Nelson, 2018; Mercer, 2019; Philp and Duchesne, 2016). It is a dynamic, multifaceted construct encompassing emotional, cognitive, and behavioral dimensions (Fredricks et al., 2004; Furlong et al., 2003; Jimerson et al., 2003). Behavioral engagement refers to on-task attention and effort; emotional engagement to attitudes and affective reactions; cognitive engagement to effortful thinking and deep processing. Reeve and Tseng (2011) proposed agentic engagement as a fourth dimension, defined as students’ constructive contribution to the flow of instruction, emphasizing how learners actively customize their learning experience. As a malleable construct, engagement predicts academic progress and indicates positive functioning (Lei et al., 2018). Self-report surveys are most frequently used due to their practicality and ability to capture less observable dimensions of engagement (Appleton et al., 2008; Dörnyei and Taguchi, 2009; Hiver et al., 2024). Although engagement has been extensively studied in general education and second language acquisition, its multidimensional structure in interpreting training remains underexplored.

The emergence of positive psychology has encouraged educational research to move beyond deficit-based perspectives and examine positive psychological resources that support learning, such as resilience, emotion regulation, and wellbeing (Seligman et al., 2009; Shoshani and Steinmetz, 2014). This trend has also reached second language acquisition (Dewaele and MacIntyre, 2014; MacIntyre and Gregersen, 2012). Recent bibliometric analyses confirm that translation and interpreting studies as a discipline has become increasingly multidisciplinary, with psychological and cognitive themes representing emerging research frontiers (Pan and Wu, 2024). Therefore, examining how positive psychological factors are associated with interpreting learning engagement represents a timely direction for interpreter education research.

Resilience, defined as the capacity to adapt positively despite adversity (Connor and Davidson, 2003), may be particularly relevant to interpreting learning, where learners frequently encounter demanding tasks and performance setbacks. Resilient individuals are more likely to recover from difficulties, sustain motivation, and maintain goal-directed behaviors (Fredrickson et al., 2003; Mansfield et al., 2016). Previous educational research has consistently demonstrated a positive relationship between resilience and learning engagement (Azila-Gbettor, 2024; Martin and Marsh, 2009; Wang and Eccles, 2012). In language learning contexts, resilience has been positively associated with motivation and negatively associated with demotivation (Kim et al., 2018, 2019). However, its associations with all four dimensions of interpreting learning engagement remain unclear.

Emotion regulation refers to the processes through which individuals influence their emotional experiences and expressions in pursuit of goals (Gross, 2015). Cognitive reappraisal (CR) and expressive suppression (ES) are the two most widely studied emotion regulation strategies (Egloff et al., 2006). CR involves reinterpretation of emotion-eliciting situations to alter emotional impact, whereas ES involves inhibiting emotional expressions (Gross and Levenson, 1993; Gross and John, 2003). Previous studies suggest that CR is generally associated with adaptive outcomes, whereas ES is more often linked to maladaptive consequences (Dryman and Heimberg, 2018; Schäfer et al., 2017). Nevertheless, findings regarding the relationship between emotion regulation and engagement remain mixed (Fried and Chapman, 2012; Kwon et al., 2018; Huang et al., 2023), possibly due to differences in learner characteristics, measurement approaches, and educational contexts.

Resilience and emotion regulation are conceptually related but operate at different levels of proximity to learning behavior. Resilience reflects a broad capacity to adapt to adversity, maintain emotional stability, and recover from setbacks (Connor and Davidson, 2003; Mansfield et al., 2016). This adaptive capacity may be expressed through flexible self-regulation: when confronted with demanding tasks, resilient learners may be better able to draw on and adjust specific strategies to manage their responses (Ge, 2025). CR is one such proximal strategy because it involves reframing the meaning of stressful experiences. Its close association with adaptability and cognitive flexibility provides a theoretical basis for treating resilience as the more distal construct and CR as the more proximal regulatory process (Liao and Hu, 2025). ES, by contrast, is a response-focused strategy that inhibits emotional expression after an emotion has arisen (Gross and John, 2003). The proposed direction from resilience to CR and ES, and then to engagement, therefore reflects a broad-resource-to-proximal-strategy account. It does not imply that resilience temporally precedes or causes emotion regulation. Because all variables were measured concurrently, reverse and reciprocal associations remain plausible.

In interpreting education, emotion regulation is especially important because learners frequently experience negative emotions associated with performance evaluation, feedback, and task difficulty. Longitudinal evidence indicates that negative emotions among interpreting students are not uniformly detrimental; their fluctuations may coincide with strategic adjustment and performance under particular conditions, such as high cognitive load or later learning stages (Jiang and Lv, 2026; Su, 2023). This provides a rationale for investigating emotion regulation as a correlate of engagement in this context and aligns with growing recognition of cognitive and psychological factors in translation and interpreting research (Pan and Wu, 2024). CR may be more strongly associated with engagement because this strategy enables learners to reinterpret errors, feedback, and performance pressure before these experiences dominate attention and motivation. By contrast, ES may be associated with cognitive costs that may be particularly relevant to the listening, memory, and reformulation demands of interpreting. Therefore, CR was expected to be more strongly associated with interpreting learning engagement than ES.

Existing research has yet to provide a comprehensive understanding of engagement in interpreting learning. Specifically, studies have often overlooked the role of agentic engagement and the multidimensional nature of engagement in this context. Findings on emotion regulation and engagement remain inconclusive, particularly regarding the distinct associations of CR and ES with different engagement dimensions. Recent research has indicated a positive association between emotion regulation and resilience (Tortosa Martínez et al., 2023), while the importance of emotions for interpreting proficiency has also been empirically demonstrated (Su, 2023). However, the associations through which resilience and different emotion regulation strategies are related to behavioral, emotional, cognitive, and agentic engagement remain underexplored. Therefore, the present study applies the theoretically informed broad-resource-to-proximal-strategy direction described above to examine a pattern of concurrent associations among resilience, CR and ES, and behavioral, emotional, cognitive, and agentic engagement. The model is an associative statistical specification rather than a causal or temporal account. The following research questions were addressed:

*Research question 1 (RQ1)*: What is the relationship between resilience, emotion regulation (CR and ES), and interpreting learning engagement?

*Research question 2 (RQ2)*: How do resilience and emotion regulation (CR and ES) predict interpreting learning engagement?

*Research question 3 (RQ3)*: Does emotion regulation (CR and ES) mediate the relationship between resilience and interpreting learning engagement?

## 2 Methods

### 2.1 Participants

To determine the required sample size, we followed Fritz and MacKinnon (2007). According to their simulation, for an indirect effect of intermediate size between small and medium tested with the bias-corrected bootstrap, a minimum of 148 participants is needed. In the present study, participants were 306 university students majoring in foreign language studies or translation and interpreting (49 males, 257 females, mean age 20.79 years), recruited using convenience sampling from five universities: Beijing International Studies University, Beijing Foreign Studies University, Shanghai International Studies University, Jimei University, and Henan Polytech University, located in major cities across China. This selection was intended to capture institutional and geographic diversity. The gender distribution was predominantly female (84.0%), which is broadly consistent with the demographic composition of foreign language and translation programs in Chinese higher education. Recruitment occurred during Consecutive Interpreting classes with instructor approval. All students present in the selected classes on the day of recruitment were invited to participate, and all agreed, yielding a 100% response rate. Inclusion criterion was: current enrolment in undergraduate/graduate interpreting courses. Exclusion criteria were: (a) incomplete responses (missing >10% of items), and (b) straight-lining responses (identical answers across an entire scale). After applying these criteria, 306 valid cases remained, with no missing data. Participants included first-year (3.3%), second-year (23.2%), third-year (13.4%), fourth-year undergraduates (27.5%), and graduate students (first-year 18.6%, second-year 11.4%, third-year 2.6%). Most were undergraduates in Foreign Language Studies (38.9%) and Translation and Interpreting (30.7%), with 21.2% in graduate programs in Translation and Interpreting (non-academic/professional track). All participants were native Chinese speakers with their second language as the foreign language for interpreting.

### 2.2 Measurement

The questionnaire collected demographic information and measures of resilience, emotion regulation, and interpreting learning engagement. Data analysis used JASP 0.17 (Love et al., 2019) to examine reliability and validity. For scales originally developed in English, the first author translated them into Chinese, and a research assistant independently back-translated them into English. Any discrepancies between the two versions were resolved through discussion to ensure semantic equivalence with the original instruments.

#### 2.2.1 Resilience

Resilience was measured using the 10-item (5-point scale) Connor-Davidson Resilience Scale (CD-RISC) (Campbell-Sills and Stein, 2007). The Cronbach’s alpha was 0.886, demonstrating satisfactory reliability. The confirmatory factor analysis (CFA), performed to assess the construct validity showed an acceptable model fit, Comparative Fit Index (CFI): 0.947, Tucker-Lewis Index (TLI): 0.928, Bollen’s Incremental Fit Index (IFI): 0.948, Root Mean Square Error of Approximation (RMSEA): 0.083, Standardized Root Mean Square Residual (SRMR): 0.040.

#### 2.2.2 Emotion regulation

The emotion regulation scale was derived from the study of Gross and John (2003) with two dimensions: CR (6 items, 7-point scale) and ES (4 items, 7-point scale). The internal consistency reliability of emotion regulation was also confirmed, Cronbach’s alpha for CR: 0.854, Cronbach’s alpha for ES: 0.824, Cronbach’s alpha for overall emotion regulation: 0.774. The CFA results also showed adequate model fit, CFI: 0.967, TLI: 0.957, IFI: 0.968, RMSEA: 0.061, SRMR: 0.049.

#### 2.2.3 Interpreting learning engagement

The Interpreting Learning Engagement Scale (ILES) (Yu and Wu, 2025) was adapted from Oga-Baldwin’s (2019) scale, which originally contained 19 items (5-point scale). The ILES removed one item related to emotional engagement because of cross-loading in the exploratory factor analysis (EFA, factor 1 loading: 0.411, factor 2 loading: 0.499) found in Yu and Wu (2025). The ILES originally consisted of 18 items: behavioral engagement (BE, 5 items), emotional engagement (EE, 3 items), cognitive engagement (CE, 5 items), and agentic engagement (AGE, 5 items). The reliability of the scale was confirmed, Cronbach’s alpha for BE: 0.904, Cronbach’s alpha for EE: 0.916, Cronbach’s alpha for CE: 0.887, Cronbach’s alpha for AGE: 0.875, Cronbach’s alpha for Engagement: 0.942. The construct of the scale was also supported by the CFA results, CFI: 0.924, TLI: 0.910, IFI: 0.925, RMSEA: 0.088, SRMR: 0.048.

### 2.3 Data collection procedure

Ethical approval was granted by the Research Ethics Committee of the authors’ university, and informed consent was obtained. All 306 students completed an online questionnaire assessing resilience, emotion regulation, and interpreting learning engagement (approx. 12 min). The Interpreting Learning Engagement Scale (ILES) items (18 items across four dimensions) used in this study overlap with those reported in a prior scale validation study (Yu and Wu, 2025). All other measures, including resilience and emotion regulation, are new to the present analysis. To assess potential common method variance (CMV), we conducted Harman’s single-factor test (Podsakoff et al., 2003). All items were entered into an exploratory factor analysis (EFA) without rotation. The first unrotated factor accounted for 29.2% of the total variance, which is below the recommended threshold of 40%, indicating that CMV is not a serious concern in the present study.

## 3 Results

### 3.1 Descriptive statistics

The descriptive statistical results are presented in Table 1. Participants exhibited relatively high levels of Resilience (3.502/5), Cognitive Reappraisal (CR) (5.115/7), Behavioral Engagement (BE) (4.029/5), Emotional Engagement (EE) (4.026/5), Cognitive Engagement (CE) (4.015/5), and Agentic Engagement (AGE) (3.485/5), with the exception of Expressive Suppression (ES) (3.651/7). These results suggest overall positive attitudes, personality traits, and emotion regulation skills.

**Table 1:** Statistical description for all study variables.

	Mean	SD	Skewness	Kurtosis
Resilience	3.502	0.638	−0.268	0.472
Cognitive reappraisal	5.115	0.844	−0.175	0.625
Expressive suppression	3.651	1.290	0.103	−0.475
Behavioral engagement	4.029	0.642	−0.488	0.529
Emotional engagement	4.026	0.768	−0.604	0.359
Cognitive engagement	4.015	0.648	−0.625	1.469
Agentic engagement	3.485	0.820	−0.078	−0.298

### 3.2 Correlation of the variables

The correlation matrix of the examined variables is presented in Table 2. It was found that all the variables except ES were significantly positively correlated with each other. ES was not significantly correlated with any of the other variables. These findings suggest that for the two emotion regulation strategies, CR rather than ES showed a positive correlation with resilience and with all four dimensions of interpreting learning engagement.

**Table 2:** Correlations among study variables.

	1	2	3	4	5	6	7
1. Resilience	–						
2. CR	0.440^***^	–					
3. ES	0.017	0.108	–				
4. BE	0.301^***^	0.356^***^	0.095	–			
5. EE	0.347^***^	0.290^***^	0.033	0.690^***^	–		
6. CE	0.315^***^	0.368^***^	0.096	0.708^***^	0.678^***^	–	
7. AGE	0.280^***^	0.268^***^	0.069	0.548^***^	0.543^***^	0.566^***^	–

CR, Cognitive Reappraisal; ES, Expressive Suppression; BE, Behavioral Engagement; EE, Emotional Engagement; CE, Cognitive Engagement; AGE, Agentic Engagement. ^***^p < 0.001.

### 3.3 Predictive effects of resilience and emotion regulation on interpreting learning engagement

Regression analyses showed that resilience and CR could predict behavioral engagement (Adjusted *R*^2^ = 14.8%, *F* (3, 302) = 18.67, *β* = 0.181, *t* = 3.076, *p* < 0.01 for resilience; *β* = 0.270, *t* = 4.553, *p* < 0.001 for CR), emotional engagement (Adjusted *R*^2^ = 14.4%, *F* (3, 302) = 16.92, *β* = 0.272, *t* = 4.578, *p* < 0.001 for resilience; *β* = 0.170, *t* = 2.843, *p* < 0.01 for CR), cognitive engagement (Adjusted *R*^2^ = 16.8%, *F* (3, 302) = 20.38, *β* = 0.192, *t* = 3.278, *p* < 0.01 for resilience; *β* = 0.277, *t* = 4.717, *p* < 0.001 for CR), and agentic engagement (Adjusted *R*^2^ = 10.7%, *F* (3, 302) = 12.01, *β* = 0.203, *t* = 3.347, *p* < 0.001 for resilience; *β* = 0.174, *t* = 2.853, *p* < 0.01 for CR). However, ES failed to predict behavioral engagement (*β* = 0.063, *t* = 1.178, *p* > 0.05), cognitive engagement (*β* = 0.062, *t* = 1.179, *p* > 0.05), emotional engagement (*β* = 0.010, *t* = 0.185, *p* > 0.05), or agentic engagement (*β* = 0.046, *t* = 0.845, *p* > 0.05). Given the unbalanced gender distribution in the sample, we included gender as a factor in the regression analyses. The results indicated that gender did not significantly predict any of the engagement dimensions (all *ps* > 0.05, see Table 3 for results adding gender as a predictor).

**Table 3:** Multiple regression results predicting four dimensions of interpreting learning engagement.

Predictors	BE	EE	CE	AGE
	*β* (SE) [95% CI]	*β* (SE) [95% CI]	*β* (SE) [95% CI]	*β* (SE) [95% CI]
Gender	0.09 (0.09) [−0.02, 0.35]	−0.03 (0.11) [−0.29, 0.16]	0.01 (0.09) [−0.16, 0.21]	−0.02 (0.12) [−0.27, 0.21]
Resilience	0.19^**^ (0.06) [0.07, 0.31]	0.27^***^ (0.07) [0.18, 0.47]	0.19^**^ (0.06) [0.08, 0.31]	0.20^**^ (0.08) [0.11, 0.41]
CR	0.27^***^ (0.05) [0.12, 0.29]	0.17^**^ (0.05) [0.05, 0.26]	0.28^***^ (0.05) [0.12, 0.30]	0.17^**^ (0.06) [0.05, 0.29]
ES	0.08 (0.03) [−0.01, 0.09]	0.01 (0.03) [−0.06, 0.07]	0.06 (0.03) [−0.02, 0.09]	0.04 (0.04) [−0.04, 0.10]
Model fit				
*R* ^2^	0.16	0.15	0.17	0.11
Adjusted *R*^2^	0.15	0.13	0.16	0.10
*F* (df_1_, df_2_)	14.84 (4, 301)	12.74 (4, 301)	15.25 (4, 301)	8.90 (4, 301)
VIF range	1.04–1.26	1.04–1.26	1.04–1.26	1.04–1.26

CR, Cognitive Reappraisal; ES, Expressive Suppression; BE, Behavioral Engagement; EE, Emotional Engagement; CE, Cognitive Engagement; AGE, Agentic Engagement. ^***^p < 0.001, ^**^p < 0.01.

### 3.4 Emotion regulation as an associative mediator between resilience and engagement

Before presenting the structural model of the hypothesized relationships between the variables, we first tested the measurement model. The factor structure of the relevant latent constructs was validated via a confirmatory factor analysis (CFA) conducted using the lavaan package (Rosseel, 2012) embedded in JASP (version 0.96.0.0). Given the ordinal nature of the Likert-scale indicators, the Diagonally Weighted Least Squares (DWLS) estimator was employed. Missing data were handled using Full Information Maximum Likelihood (FI-MLE). The results indicated that the measurement model was well supported: CFI = 0.964, TLI = 0.961, RMSEA = 0.055 [90% CI lower bound: 0.048, 90% CI upper bound: 0.062], SRMR = 0.051.

For the structural model, the Diagonally Weighted Least Squares (DWLS) estimator was employed. The Bollen–Stine bootstrap test with 1,000 resamples was used to evaluate global model fit. Missing data were addressed using Full Information Maximum Likelihood (FI-MLE), and 95% confidence intervals were reported. The associations of CR and ES with resilience and interpreting learning engagement are presented in Table 4. Consistent with the correlation analysis, ES showed no significant association with the four dimensions of engagement after accounting for resilience. However, CR was positively associated with resilience and all four engagement dimensions (behavioral, emotional, cognitive, and agentic engagement), suggesting that resilience and CR share overlapping variance in predicting engagement outcomes. The proportion of the total effect of resilience on each engagement dimension that was mediated by CR (i.e., the proportion mediated, PM) was calculated as the indirect effect divided by the total effect. The PM for behavioral engagement was 50.0%, followed by cognitive engagement (47.2%) and agentic engagement (34.4%). The PM for emotional engagement was comparatively lower, at 25.6%. These findings indicate that CR, rather than ES, is more closely linked to engagement in interpreting learning.

**Table 4:** Emotion regulation as an associative mediator between resilience and engagement.

	Total effects	Direct effects	Indirect effects via CR	Indirect effects via ES
	[95%CI], SE	[95%CI], SE	[95%CI], SE	[95%CI], SE
Re → BE	0.34^***^ [0.23, 0.45], 0.06	0.17^*^ [0.03, 0.31], 0.07	0.17^***^ [0.09, 0.24], 0.04	0.01 [−0.01, 0.02], 0.01
Re → EE	0.39^***^ [0.28, 0.49], 0.06	0.29^***^ [0.15, 0.43], 0.07	0.10^**^ [0.02, 0.17], 0.04	0.00 [−0.01, 0.01], 0.00
Re → CE	0.36^***^ [0.25, 0.48], 0.06	0.18^*^ [0.04, 0.33], 0.07	0.17^***^ [0.09, 0.25], 0.04	0.01 [−0.01, 0.02], 0.01
Re → AGE	0.32^***^ [0.20, 0.44], 0.06	0.21^**^ [0.06, 0.36], 0.07	0.11^**^ [0.03, 0.19], 0.04	0.00 [−0.01, 0.01], 0.01

Re, Resilience; CR, Cognitive Reappraisal; ES, Expressive Suppression; BE, Behavioral Engagement; EE, Emotional Engagement; CE, Cognitive Engagement; AGE, Agentic Engagement. ^*^p < 0.05, ^**^p < 0.01, ^***^p < 0.001.

## 4 Discussion and conclusion

Engagement is especially pertinent for students facing stressful or high-risk circumstances (Ungar and Liebenberg, 2013), making it especially relevant in interpreting learning. Yet, empirical research on interpreting learning engagement remains scarce. To our knowledge, this study is the first to empirically examine the multivariate associations of resilience and emotion regulation with all four dimensions of interpreting learning engagement. The findings reveal that resilience and CR were positively associated with and served as predictors of behavioral, emotional, cognitive, and agentic engagement. Furthermore, in the relationship between resilience and interpreting learning engagement, CR rather than ES served as a significant associative mediator. Because the data are cross-sectional, these findings do not establish temporal precedence or causal mediation.

### 4.1 Associations between resilience and emotion regulation

Prior research has reported associations between resilience and emotion regulation, but it has not established a single temporal ordering between them. The theoretical direction adopted here rests on the distinction between resilience as a relatively distal adaptive resource and CR and ES as more proximal regulatory processes. In the current data, resilience was positively associated with CR (*r* = 0.440, *p* < 0.001), but not with ES. This pattern is consistent with previous reports linking greater CR use with higher resilience, and greater ES use with lower resilience (Mouatsou and Koutra, 2023; Zarotti et al., 2020). Adaptive strategies such as CR are linked to positive health outcomes, mental wellbeing, and competencies that are important for educational success and resilience, while maladaptive strategies like ES may hinder such outcomes (Chervonsky and Hunt, 2019; Salmela-Aro and Read, 2017; Troy and Mauss, 2011). However, the present correlation is equally compatible with resilience being associated with CR, CR being associated with resilience, or a reciprocal relationship. It should therefore be interpreted as shared covariation rather than a directional process. Longitudinal studies with repeated measures are needed to compare these alternatives.

### 4.2 Resilience and interpreting learning engagement

Linear regression analyses reveal that resilience significantly predicts student interpreters’ engagement across all four dimensions. The correlational analysis demonstrates a significant positive relationship between resilience and the four dimensions of interpreting learning engagement. These results align with previous studies highlighting the association of resilience with learning engagement. For instance, Xu and Feng (2024) found that resilience was strongly correlated with behavioral engagement (*r* = 0.708), emotional engagement (*r* = 0.572), and cognitive engagement (*r* = 0.598) in L2 learning. Similarly, Azila-Gbettor (2024) found that academic resilience significantly predicted engagement among science students (*β* = 0.378, *p* = 0.001), while Liu et al. (2022) also observed a significant association between academic resilience and language learning engagement (*r* = 0.59, *p* < 0.001). These consistent findings underscore resilience as a key factor linked to sustained engagement across educational contexts. Resilient students are better prepared to overcome challenges and stay intellectually engaged (Reyes et al., 2015), and their capacity to endure adversity (Li and Yang, 2016) further supports resilience a strong predictor of engagement in interpreting. The present cross-sectional results should be interpreted more narrowly: learners reporting higher resilience also tended to report higher behavioral, emotional, cognitive, and agentic engagement. They do not show that resilience produces or sustains engagement.

### 4.3 The associative mediation of emotion regulation in the relationship between resilience and interpreting learning engagement: CR as a mediator rather than ES

This study further contributes by examining how emotion regulation predicts and serves as an associative mediator between resilience and interpreting learning engagement. Existing literature has identified the role of emotion regulation in learning engagement (Morrish et al., 2018; Dang et al., 2025), but there is limited understanding of how different emotion regulation strategies might be uniquely associated with engagement (Santos et al., 2021). This study found that CR was positively associated with, and predicted, all four engagement dimensions, whereas ES showed no significant association or predictive power. These results echo Santos et al. (2021), who, in their study of 1,713 Portuguese participants, found adaptive emotion regulation strategies like CR and Refocusing on Planning to be moderately correlated with engagement. The reinterpretive orientation of CR is consistent with a more constructive appraisal of learning challenges, but the present data do not show that CR changes motivation, attention, or engagement. ES did not demonstrate significant associations with resilience or any engagement dimension in this sample. This finding suggests that, unlike CR, ES may not represent a relevant mechanism linking resilience and engagement among interpreting learners in this sample. Although previous research has associated ES with less adaptive psychological outcomes (Dryman and Heimberg, 2018; Schäfer et al., 2017), its associations may vary across learner characteristics, contextual demands, and outcomes. The nonsignificant findings here should not be interpreted as evidence that ES is detrimental to interpreting learning engagement. Future research should examine whether its associations vary across contexts and over time.

Within the specified model, CR accounted for a partial associative mediation between resilience and interpreting engagement, whereas ES did not. This associative decomposition is compatible with the proposed ordering but does not establish temporal precedence or a causal process. The result instead identified a pattern of shared variance that could be evaluated more rigorously in longitudinal or experimental research. Accordingly, CR-focused pedagogical practices should be treated as hypotheses for intervention testing rather than as established methods for increasing engagement.

### 4.4 Limitations and future directions

This study has several limitations. First, potential gender- and age-related differences were not explored, despite evidence suggesting such effects (Isaacowitz, 2022; Santos et al., 2021). Future studies should examine how these factors may moderate the relationships among key variables. Second, only CR and ES were examined; other strategies such as Acceptance, Putting into Perspective, and Rumination may also be associated with engagement (Santos et al., 2021). Including a broader range would allow a more nuanced understanding of emotion regulation in interpreter education. Third, engagement is dynamic and fluctuates over time (Mercer, 2019), yet this study relied on cross-sectional retrospective self-reports. Future longitudinal research should track engagement over extended periods. Fourth, sole reliance on self-report questionnaires may introduce common-method variance (Podsakoff et al., 2003). Fifth, the cross-sectional design precludes causal inferences. Future studies could further use longitudinal and experimental designs to explore how emotion regulation influences interpreting teaching and learning. Sixth, the convenience sample limits generalizability beyond Chinese foreign language majors. Further data collection from other countries and areas could be useful for generalization of the present study results.

### 4.5 Implications for interpreting training

The positive associations of resilience and CR with engagement suggest that CR-focused activities may be worth evaluating in interpreter education. Candidate practices for future intervention testing include pre-task CR prompts, in which students reframe anticipated challenges before interpreting; post-mock reflective journals using guided questions such as “What can I learn from this error?”; and structured peer-feedback scripts that focus on specific behaviors rather than global judgments. The present cross-sectional findings do not establish changes in engagement or learning outcomes attributable to these activities; their pedagogical value requires longitudinal or experimental evaluation.

## Data Availability

The raw data supporting the conclusions of this article will be made available by the authors, without undue reservation.
