# Supplementary material for: Resilience, emotion regulation, and interpreting learning engagement: cognitive reappraisal as a mediator rather than expressive suppression
Source: Front Psychol. 2026 Sep 3;17:1916373. doi: 10.3389/fpsyg.2026.1916373 (PMC13582544; doi:10.3389/fpsyg.2026.1916373)
Supplement: Supplementary file 1 [file Data_Sheet_1.pdf]

# *Supplementary Material*

**Supplementary Table 1: ILES (English Version)**

| **No.** | **Sub-construct** | **ILES Items** |
|---|---|---|
| 1 | BE1 | I participated in the activities of this semester's interpreting classes |
| 2 | BE2 | I paid attention to my interpreting teacher this semester |
| 3 | BE3 | I listened very carefully in the interpreting classes this semester |
| 4 | BE4 | I tried to do well in this semester's interpreting classes |
| 5 | BE5 | I worked as hard as I could in this semester's interpreting classes |
| 6 | EE2 | I enjoyed this semester's interpreting classroom activities |
| 7 | EE3 | I was interested in the materials of this semester's interpreting classes |
| 8 | EE4 | This semester's interpreting classes were fun |
| 9 | CE1 | I applied the interpreting skills and techniques that I learned in class to practicing interpreting this semester |
| 10 | CE2 | I tried to fully comprehend the exact messages in the source speeches in this semester's interpreting classes |
| 11 | CE3 | I tried to fully deliver the exact messages in the target Language in this semester's interpreting classes |

| | | |
|---|---|---|
| 12 | CE4 | I thought about the teacher's and peer students' feedback on my interpreting performance in this semester's interpreting classes |
| 13 | CE5 | I thought about the strengths and weaknesses of my interpreting performance in this semester's interpreting classes |
| 14 | AGE1 | I let my teacher know what I considered difficult in practicing interpreting in this semester's interpreting classes |
| 15 | AGE2 | I let my teacher know what I was satisfied with the most about my interpreting practice in this semester's interpreting classes |
| 16 | AGE3 | I expressed my opinions in the interpreting classes this semester. |
| 17 | AGE4 | I asked questions to help myself learn more knowledge and skills of interpreting in this semester's interpreting classes |
| 18 | AGE5 | When I ran into difficulties in practicing interpreting in this semester's interpreting classes, I asked my teacher for advice |

**Supplementary Table 2: ILES (Chinese Version)**

| **No.** | **Sub-construct** | **ILES Items** |
|---|---|---|
| 1 | BE1 | 我认真参与了这学期口译课的课堂口译学习。 |
| 2 | BE2 | 我在这学期口译课上专心地听老师讲课。 |
| 3 | BE3 | 我在这学期口译课上仔细听其他同学发言。 |
| 4 | BE4 | 我在这学期口译课上努力表现好。 |

| | | |
|---|---|---|
| 5 | BE5 | 我在这学期口译课上尽我所能投入学习。 |
| 6 | EE2 | 我很喜欢参与这学期口译课的课堂活动。 |
| 7 | EE3 | 我对这学期口译课的教学材料感兴趣。 |
| 8 | EE4 | 这学期的口译课很有趣。 |
| 9 | CE1 | 这学期口译课上的练习我运用了所教授的口译技巧和策略。 |
| 10 | CE2 | 这学期口译课上我尝试充分理解源语中的确切信息。 |
| 11 | CE3 | 这学期口译课上我尝试用目标语充分译出源语的确切信息。 |
| 12 | CE4 | 这学期口译课上我就老师和同学对我课上的口译表现反馈进行了思考。 |
| 13 | CE5 | 这学期口译课上我就自己在课上的口译表现的优缺点进行了思考。 |
| 14 | AGE1 | 这学期我让老师知道我在课上练习口译时遇到哪些困难。 |
| 15 | AGE2 | 这学期我让老师知道我在课上练习口译时自己感到最满意的部分。 |
| 16 | AGE3 | 这学期口译课上我表达过自己的想法。 |
| 17 | AGE4 | 这学期我通过提问来帮助自己学得更多口译相关的知识和技能。 |
| 18 | AGE5 | 这学期当我在口译练习中遇到困难时，我会主动求助于老师。 |

**Supplementary Table 3: Refined version of the CD-RISC (Campbell-Sills & Stein 2007) (English Version)**

| No. | Resilience Items |
|---|---|
| 1 | I’m able to adapt to change. |
| 2 | I can deal with whatever comes. |
| 3 | I try to see the humorous side of problems. |
| 4 | Coping with stress can strengthen me. |
| 5 | I tend to bounce back after illness or hardship. |
| 6 | I can achieve goals despite obstacles. |
| 7 | I can stay focused under pressure. |
| 8 | I’m not easily discouraged by failure. |
| 9 | I think of myself as a strong person. |
| 10 | I can handle unpleasant feelings. |

**Supplementary Table 4: Refined version of the CD-RISC (Campbell-Sills & Stein 2007) (Chinese Version)**

| No. | Resilience Items |
|---|---|
| 1 | 我能够适应变化。 |
| 2 | 我能应付任何情况。 |
| 3 | 我尝试看到问题有趣的一面。 |

| | |
|---|---|
| 4 | 应对压力会令我变得更强。 |
| 5 | 遭遇疾病或苦难后我会重振旗鼓。 |
| 6 | 尽管遇到障碍，我仍能实现目标。 |
| 7 | 在压力下我能保持专注。 |
| 8 | 失败不会轻易让我气馁。 |
| 9 | 我认为自己是个坚强的人。 |
| 10 | 我能处理不愉快的情绪。 |

**Supplementary Table 5: The Emotion Regulation Questionnaire (ERQ) (Gross & John 2003) (English Version)**

| No. | Sub-construct | ERQ Items |
|---|---|---|
| 1 | Cognitive Reappraisal (CR) | When I want to feel more positive emotion (such as joy or amusement), I change what I'm thinking about. |
| 2 | Expressive Suppression (ES) | I keep my emotions to myself. |
| 3 | Cognitive Reappraisal (CR) | When I want to feel less negative emotion (such as sadness or anger), I change what I'm thinking about. |
| 4 | Expressive Suppression (ES) | When I am feeling positive emotions, I am careful not to express them. |
| 5 | Cognitive Reappraisal (CR) | When I'm faced with a stressful situation, I make myself think about it in a way that helps me stay calm. |
| 6 | Expressive Suppression (ES) | I control my emotions by not expressing them. |

| | | |
|---|---|---|
| 7 | Cognitive Reappraisal (CR) | When I want to feel more positive emotion, I change the way I'm thinking about the situation. |
| 8 | Cognitive Reappraisal (CR) | I control my emotions by changing the way I think about the situation I'm in. |
| 9 | Expressive Suppression (ES) | When I am feeling negative emotions, I make sure not to express them |
| 10 | Cognitive Reappraisal (CR) | When I want to feel less negative emotion, I change the way I'm thinking about the situation. |

**Supplementary Table 6: The Emotion Regulation Questionnaire (ERQ) (Gross & John 2003) (Chinese Version)**

| **No.** | **Sub-construct** | **ERQ Items** |
|---|---|---|
| 1 | Cognitive Reappraisal (CR) | 当我想感受一些积极的情绪（如快乐或高兴）时，我会改变自己思考问题的角度。 |
| 2 | Expressive Suppression (ES) | 我不会表露自己的情绪。 |
| 3 | Cognitive Reappraisal (CR) | 当我想少感受一些消极的情绪（如悲伤或愤怒）时，我会改变自己思考问题的角度。 |
| 4 | Expressive Suppression (ES) | 当感受到积极情绪时，我会很小心的不让它们表露出来。 |
| 5 | Cognitive Reappraisal (CR) | 在面对压力情境时，我会使自己以一种有助于保持平静的方式来考虑它。 |
| 6 | Expressive Suppression (ES) | 我控制自己情绪的方式是不表达它们。 |
| 7 | Cognitive Reappraisal (CR) | 当我想多感受一些积极的情绪时，我会改变自己对情境的考虑方式。 |

| | | |
|---|---|---|
| 8 | Cognitive Reappraisal (CR) | 我会通过改变对情境的考虑方式来控制自己的情绪。 |
| 9 | Expressive Suppression (ES) | 当感受到消极的情绪时，我确定不会表露它们。 |
| 10 | Cognitive Reappraisal (CR) | 当我想少感受一些消极的情绪时，我会改变自己对情境的考虑方式。 |
